# The effect of nitrogen atmosphere during post-curing on cytotoxicity, polishability, flexural strength, and surface hardness of 3D-printed denture bases: an in vitro study

**DOI:** 10.1007/s10856-026-07006-5

**Published:** 2026-01-26

**Authors:** Karoline Gladrow, Alexey Unkovskiy, Jamila Yassine, Nora Gaertner, Ievgeniia Topolniak, Nico Henning, Franziska Schmidt

**Affiliations:** 1 Charité—Universitätsmedizin Berlin, Corporate Member of Freie Universität Berlin, Humboldt-Universität zu Berlin, and Berlin Institute of Health, Dental Materials and Biomaterial Research, Department of Prosthodontics, Geriatric Dentistry and Craniomandibular Disorders, Berlin, Germany; 2 Department of Dental Surgery, Sechenov First Moscow State Medical University, Moscow, Russia; 3 Bundesanstalt für Materialforschung und - prüfung (BAM), Berlin, Germany

## Abstract

3D printing is increasingly utilized in dentistry. Compared to traditional manufacturing methods, 3D printing provides advantages such as faster production times and the ability to create complex structures. Although biocompatible materials are available, many are only suitable for temporary applications. This study examines the impact of nitrogen-aided post-processing on the mechanical properties and cytotoxicity of 3D-printed denture bases, with the hypothesis that this post-processing will enhance material properties and decrease cytotoxicity. Specimens were fabricated from V-print dentbase (Voco GmbH, Cuxhaven, Germany) and post-processed either in nitrogen or air. The specimens were categorized into aged and non-aged groups. For comparison, specimens made from milled material were utilized. Vickers hardness, flexural strength, polishability, cytotoxicity, and degree of conversion were then assessed for all groups. The data were analyzed using a one-way ANOVA and Tukey HSD test for multiple comparisons, with a significance threshold of *p* < 0.05. Post-curing with nitrogen improved the degree of conversion, surface hardness, and biocompatibility of 3D-printed dental materials, confirming reduced cytotoxicity without impairing mechanical properties. Nitrogen increased polymerization and decreased harmful monomers, making it ideal for clinical applications in contact with the oral mucosa. Optimizing post-processing steps, such as curing in nitrogen, enhances biocompatibility while maintaining strength and hardness, ensuring better patient care in dental applications.

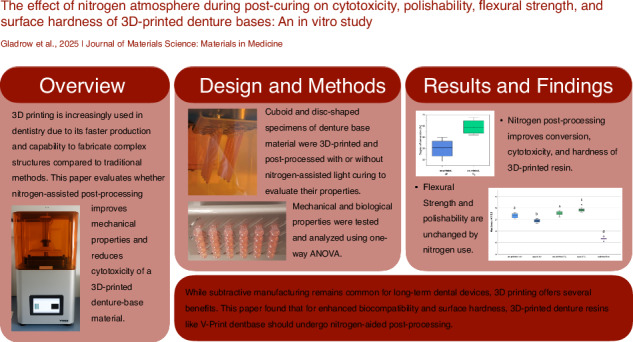

## Introduction

3D-printed devices find widespread applications in various fields of dentistry. Whether it is for planning or study models, drilling or cutting guides for autotransplantation in Endodontics, or prototypes of surgical tools [1, 2]. However, when it comes to objects meant to remain in the oral cavity for a prolonged period, subtractive manufacturing still seems to be the prevalent choice. This process is not only costly, time-consuming, and harmful to the environment due to its wastefulness, but it is also very limited, as it cannot achieve high complexity or hollow spaces [2–4]. On the other hand, additive manufacturing can achieve sub-micron resolution (depending on technique) [5], allows simultaneous printing with different materials, and the creation of hollow spaces, enabling the printing of complex structures [2]. To leverage these advantages, 3D printed materials must be biocompatible and possess mechanical properties comparable to those of materials used in subtractive manufacturing.

3D printing of clear aligners, splints, and customized brackets has already revolutionized the field of orthodontics [6]. However, dentures account for one of the largest shares in polymer processing regarding dental appliances. Therefore, the 3D printing of denture bases and the ongoing improvement of this process are highly significant. Currently, most dentures are made by modeling the shape of the future denture using wax, then creating a negative of that shape in plaster. This negative is then refilled with denture acrylic and placed in a pressure vessel for an air-bubble-free hardening process before being removed from the plaster for post-processing, such as polishing. By 3D printing the final shape in resin, the manufacturing of wax models and negatives can be avoided, significantly speeding up the manufacturing process and saving materials, energy, and time. While several biocompatible materials are available on the market for 3D printing, most are designed for relatively short-term, temporary applications, particularly concerning gingiva contact. More research and data evaluation are necessary before 3D-printed denture bases can become standard in dental practice.

The 3D printing of light-sensitive resin can be performed using various techniques, all of which utilize the photopolymerization of liquid resins in a vat. This process, commonly referred to as vat photopolymerization, is initiated differently based on the type of printer and technique used. It incorporates a vat filled with light-sensitive resin, which is selectively cured layer by layer. Depending on the printer, different technologies such as stereolithography (SLA) - where resin is cured by a laser point-by-point - or digital light processing (DLP), which employs a light projector and micromirrors to cure entire layers at once, enable varying fabrication durations [2, 7]. The latter technique was employed to create the specimens for this study.

After 3D printing objects using these methods, additional post-processing is always necessary; this includes at least cleaning the objects by washing them in isopropanol or a similar cleaning solution, further post-curing, and removing support structures [8]. Different methods are available for post-curing, each with varied atmospheres, temperatures, and light sources, which may all influence the final properties of the printed part. To utilize 3D printing materials for denture bases or similar long-term dental devices, it is essential to achieve a balance between mechanical and biocompatible properties. Existing research suggests that the elution of monomers, associated with cytotoxicity, mutagenicity, and hyposensitivity, may be reduced by altering the post-processing following 3D printing [9, 10]. The mechanical properties and durability of the 3D printed parts can remain nearly unchanged or potentially be enhanced based on the type and duration of the post-processing techniques [10–12]. To increase biocompatibility, the number of cytotoxic monomers should be minimized. This can be accomplished through a higher degree of conversion [13, 14], thereby decreasing the potential health risks in photopolymer resin-based 3D printed parts. This step may most importantly enhance the safety of patients, technicians, and practitioners.

With nitrogen displacing oxygen from free radicals, no unoccupied bonds remain on the surface after post-processing [15, 16]. Thus, post-curing 3D printed resins in a nitrogen-filled chamber could enhance biocompatibility and other mechanical properties influenced by the degree of conversion.

A recent study by Zinelis et al. [17], which examined the effect of a nitrogen atmosphere on the mechanical properties of 3D-printed aligners, found no significant difference in those properties.

On the other hand, Oh et al. [10] demonstrated effects on flexural strength, surface hardness, and cell viability by modifying washing solution temperature and washing time, suggesting a potential enhancement of material properties through changes in post-processing.

The rapid, in-house production of dentures is undoubtedly the future of dental care, as time is extremely valuable and many unnecessary intermediate steps, such as mailing routes, model fabrication, and wax-ups, can be eliminated [18]. To explore additional post-processing procedures and leverage their effects on resins, further data, evaluation, and literature must be provided. Therefore, this study aims to evaluate the effect and use of nitrogen-aided post-processing on polishability, flexural strength, Vickers hardness, and cytotoxicity of a 3D printable denture-base material, also considering a further change in these properties after aging. The study hypothesizes that post-processing with nitrogen would decrease cytotoxicity while enhancing the mechanical properties of the material, minimizing the deterioration of properties after aging.

## Materials and methods

**Fig. 1:**
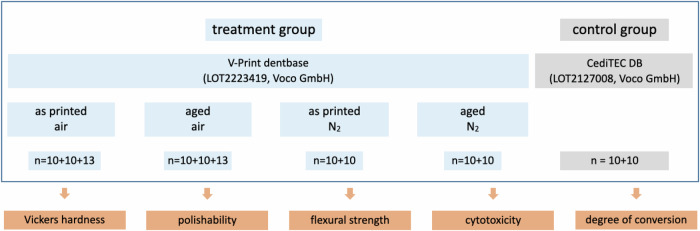
Study design

In this study a 3D printing resin, V-Print dentbase (Voco GmbH, Cuxhaven, Germany) and a millable PMMA based denture material CediTEC DB (Voco GmbH, Cuxhaven, Germany) were used (Table 1). Figure 1 shows the study design.

**Table 1:** Employed materials

Group	Commercial name	Supplier	Lot No.	Composition
Additive	V-print dentbase	Voco GmbH, Germany	LOT2223419	UDMA, BIS-EMA, TEGDMA, Diphenylphophinoxid
Subtractive	CediTEC DB	Voco GmbH, Germany	LOT2127008	>88% PMMA, < 1% coloring pigments

### Production of specimens

**Fig. 2:**
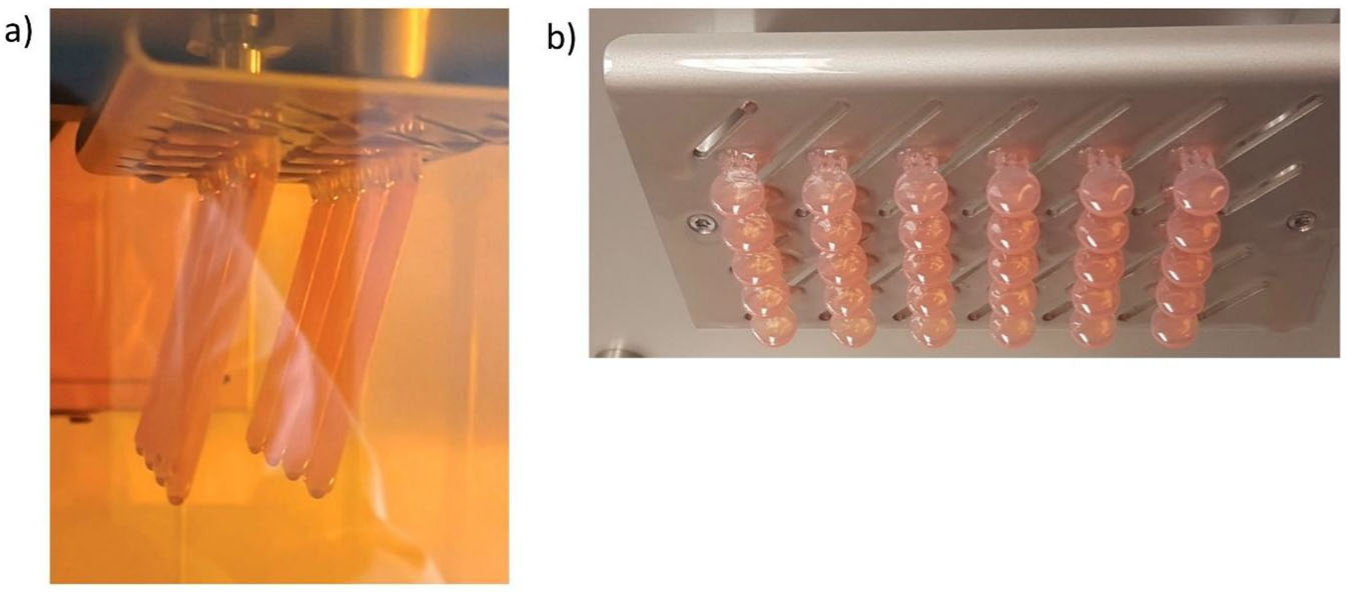
**a** Cuboid specimens inside the 3D printer, **b** cylindric specimens on building platform

Using Autodesk Netfabb, a standard tessellation language (STL) file was created for printing cuboid specimens measuring 64 × 10 x 3.3 mm, with a support structure holding them at a 105° angle. The Printing settings were set to a layer thickness of 50 µm, a support wall thickness of 300 µm, and a raster size of 1.0 mm. The support was designed to be built with a critical angle of 25°, a noncritical angle of 40°, and a minimal area of 0.1 cm^2^. The support structure held the specimens at a distance of 8.0 mm from the platform. 60 test specimens were printed using a 3D printer (SolFlex 170, Voco GmbH, Cuxhaven, Germany) from the denture base material (“additive”, V-Print dentbase, Table 1). For reference, 20 PMMA specimens (“subtractive”, CediTEC DB, Table 1) with the same dimensions were milled. For the cytotoxicity and degree of conversion test, specimens of both materials were manufactured equally in a disc shape with a 10 mm diameter and a height of 1.0 mm. Figure 2a,b shows both shapes immediately after printing, before post-processing.

All 3D-printed specimens were rinsed with isopropanol and then washed in an ultrasonic bath with isopropanol for 3 minutes as the preliminary cleaning cycle, followed by final cleaning in fresh isopropanol in an ultrasonic bath for 2 minutes. After drying the specimens with an air syringe, they were divided into two groups: Half were light-cured (Otoflash G171, NK Optik Sales GmbH, Baierbrunn, Germany) without nitrogen, following the manufacturer’s instructions, using 2000 flashes for the first cycle, the specimens were allowed to cool with an open lid for two minutes, then turned over and finished with another 2000 flashes. The support structures were removed after completing the post-processing to prevent deformation. For the second group, the light curing chamber was flooded with nitrogen for 30 seconds before starting the first cycle of 2000 flashes, during which the chamber was maintained at 1 bar of nitrogen for an additional 60 seconds. This process was repeated after the specimens were allowed to cool with an open lid for 2 minutes and turned over.

From each 3D-printed group and shape, 10 specimens were aged in artificial saliva (aldiamed gel, Certmedia International GmbH, Aschaffenburg, Germany) at 37 °C ± 1 for 24 ± 2 h.

### Physical characterization and testing

#### Polishability

To test the polishability, 10 cuboid specimens of each of the three non-aged groups were polished by hand. Each specimen was primed using grinding paper (Corund SC 180, Hager & Werken GmbH & Co. KG, Duisburg, Germany) for 45 seconds and subsequently, twice for 45 seconds with a 220-grain size, changing the grinding paper in between sets. This was followed by brushing with a coarser (Calibris, C.& L.E. Attenborough Ltd., Nottingham, UK) and finer brush (54130, VISIONDental GmbH, Oldenburg, Germany) on the brushing machine (Poliereinheit PE5, Degussa AG, Hanau, Germany) with 1500 rotations per minute using pumice slurry (ERNST HINRICHS Dental GmbH, Goslar, Germany). Polishing was finalized using a soft clothed wheel (TopDent Leinen-Polierschwabbel Kunststoffkern 100 mm, Kentzler-Kaschner Dental GmbH, Ellwangen/ Jagst, Germany) and polishing paste (Universal Polishing Paste, Ivoclar Vivadent AG; Schaan, Liechtenstein). The polishability was assessed by determining the surface roughness of all specimens. For this purpose, all specimens were tested at three different positions using an optical measuring system (InfiniteFocus, Alicona Imaging GmbH, Raaba/ Graz, Austria). This system utilized software (Alicona Imaging GmbH, Raaba/Graz, Austria) to analyze 3D images captured at a 20x magnification, calculating the maximum height (Sz) and arithmetic mean of the heights (Sa) over a measuring area of 0.323 mm² with a cutoff (λc) of 0.8 mm. The determined values were then compared between groups.

#### Mechanical properties

For the three-point bending test, cuboid specimens of all groups, including the two aged ones were prepared according to DIN EN ISO 20796-1. All specimens were polished plane-parallel using metallographic grinding papers (P500, Hermes Schleifwerkzeuge GmbH & Co.KG, Hamburg, Germany, P800, P1200, Buehler, ITW Test & Measurement GmbH, Leinfelden-Echterdingen, Germany) until a height of 3.3 ± 0.2 mm, with a variance of 0.02 mm maximum for each specimen was achieved. To determine the flexural strength, a three-point bending test was performed using the Zwick Roell universal testing machine Z010 (Z010, ZwickRoell GmbH & Co. KG, Ulm, Germany), with a pre-load of 1 N and a test speed of 5 mm/min.

Vickers hardness was tested on the same specimens as the flexural strength, at HV 0.3 and a 20x magnification using a microhardness tester (ATM Qness GmbH, Mammelzen, Germany).

#### Degree of conversion

The chemical composition of the uncured V-print dentbase and corresponding 3D printed specimens were investigated utilizing FTIR in attenuated total reflectance (ATR) mode using a Nicolet 8700 FTIR spectrometer (Thermo Fisher Scientific Inc., Massachusetts, USA). FTIR spectra were recorded with 32 scans at 4 cm^-1^ resolution in the range of 4000 to 450 cm^-1^. For each postprocessing procedure, spectra were acquired from two different samples in two distinct areas of the sample. Three samples of the unpolymerized resin were measured. Only the unaged samples were characterized.

The degree of double-bond conversion of acrylate groups (DC) was determined using the following equation:

$${DC}=\left[1-\frac{{\left({A}_{{MA}}/{A}_{1608}\right)}_{{polymerized}}}{{\left({A}_{{MA}}/{A}_{1608}\right)}_{{unpolymerized}}}\right]\times 100 \%$$

where *A*_*MA*_ is the peak height assigned to methacrylate groups, either the peak found at 1320 cm^-1^ corresponding to the stretching νC-O, or the peak at 1637 cm^-1^ for the aliphatic νC=C groups. As an internal reference for comparing different samples, we used a peak height of 1608 cm^-1^ for the aromatic νC=C groups present in BIS-EMA. The absorption of aromatic νC=C was chosen as a reference based on the assumption that the number of these bonds remains constant during the photopolymerization process. The baseline points at 1591 and 1650 cm^-1^ were utelized to determine the peak heights at 1608 and 1637 cm^-1^. Additionally, 1350 and 1209 cm^-1^ served as baseline points for detecting the peak height at 1320 cm^-1^. Baseline points were selected to minimize the influence of the spectrum background and neighboring peaks on the height of the peak of interest.

### Biocompatibility

To perform the indirect cytotoxicity test, extracts of all groups’ specimens had to be prepared according to the standard DIN EN ISO 10993, part 5 and part 12. For this purpose, the cell culture medium for L929 cells was produced using 88 ml Dulbecco’s Modified Eagle’s Medium (DMEM) (Gibco by Thermo Fisher Scientific Inc., Massachusetts, USA), 10 ml fetal bovine serum (FBS, Gibco by Thermo Fisher Scientific Inc., Massachusetts, USA), 1 ml Minimum Essential Medium Non-Essential Amino Acids solution (PAN-Biotech GmbH, Aidenbach, Germany) and 1 ml Penicillin/ Streptomycin (PAN-Biotech GmbH, Aidenbach, Germany). For each group, seven disc specimens were disinfected in 70% Ethanol (Carl Roth GmbH + Co. KG, Karlsruhe, Germany) using sterile tweezers, dried on a paper towel, and after complete drying were put in 2.2 ml cell culture medium in a 15 ml Falcon Tube (Falcon by Corning Incorporated, Corning, USA) for extraction. The tubes were then sealed, using parafilm (Bemis Curwood Parafilm M Laboratory Film, Bemis Company Inc., Neenah, USA) and put in a vertical holder on a shaker (KS 260 basic, IKA-Werke GmbH & Co. KG, Staufen, Germany) inside an incubator at 37 ± 1 °C for 24 ± 2 h. Then, specimens were removed from the tubes using sterile tweezers. The resulting extracts were used for the following cytotoxicity tests.

To obtain cells for the test, cryopreserved cells were thawed, seeded, and subcultured twice. Then, 1 × 10^4^ cells per well were pipetted into a 96-well plate, so eight groups (five for each specimen group and a negative, as well as a positive control group and a blank) could be tested. The well plate was incubated (BB 6060 CU, Heraeus Holding GmbH, Hanau, Germany) in standard medium at 37 ± 1 °C in humid atmosphere with 5% CO_2_ for 24 ± 2 h. After that, the medium was aspirated carefully without touching the cells, and 100 µl of the previously prepared sample extracts were added to 9 wells for each group. For the negative control group, 100 µl fresh medium, the positive control group, 10 µl DMSO (Thermo Fisher Scientific Inc., Massachusetts, USA) and 90 µl medium, and for the blank 100 µl medium, which was incubated exactly as the extracts were, were added to nine wells each. The well plate was then incubated for another 24 ± 2 h at 37 ± 1 °C.

For the analysis using PrestoBlue (PrestoBlue Cell Viability Reagent, Invitrogen by Thermo Fisher Scientific Inc., Massachusetts, USA) the medium of each well was again aspirated and discarded. Then, 90 µl RPMI 1640 medium (Gibco by Thermo Fisher Scientific Inc., Massachusetts, USA) was added to each well, after turning off the lights, 10 µl PrestoBlue (PrestoBlue Cell Viability Reagent, Invitrogen by Thermo Fisher Scientific Inc., Massachusetts, USA) were added to each well. The well plate was shaken in the plate reader (BMG LABTECH GmbH, Ortenberg, Germany) for 5 seconds on low setting, before wrapping it in aluminum foil, to shield it from light, and then incubated for 2 hours at 37 ± 1 °C. Finally, the absorption was measured at 570 nm and 600 nm three times using the plate reader.

To calculate the percent reduction values for each group, the following equation was used:

$${Percent\; Reduction\; of\; PrestoBlue\; Reagent}=\frac{\left(O2\cdot A1\right)-\left(O1\cdot A2\right)}{\left(R1\cdot N2\right)-\left(R2\cdot N1\right)}\cdot 100$$

- O1=molar extinction coefficient of oxidized PrestoBlueTM reagent at 570 nm=80586
- O2=molar extinction coefficient of oxidized PrestoBlueTM reagent at 600 nm=117216
- R1= molar extinction coefficient of reduced PrestoBlueTM reagent at 570 nm=155677
- R2= molar extinction coefficient of reduced PrestoBlueTM reagent at 600 nm=14652
- A1=absorbance of test wells at 570 nm
- A2=absorbance of test wells at 600 nm
- N1=absorbance of media only wells at 570 nm
- N2=absorbance of media only wells at 600 nm

The ground state of PrestoBlue reagent is the oxidized form, Resazurin, which has an absorption maximum at 600 nm. The glycolysis and citrate cycle of living cells reduce Resazurin to Resorufin, exhibiting an absorption maximum at 570 nm. Therefore, the reduction serves as an indicator of cell viability. The resulting reduction values for all groups indicate the amount of surviving cells.

To perform the direct cytotoxicity test, a concentration of 5 × 10^4^ cells per well were pipetted into a 24-well plate, so eight groups (five for each specimen group and a negative, as well as a positive control group and a blank) could be tested. The well plate was put in an incubator (BB 6060 CU, Heraeus Holding GmbH, Hanau, Germany) at 37 °C ± 1 for 24 h ± 2. After that, the medium was aspirated carefully without touching the cells, and the cell layer was checked under the microscope before carefully placing one cylindrical specimen on the cell layers of 3 wells for each group. For the negative control group, titanium discs (10 mm diameter, height 2 mm), the positive control group, 52 µl DMSO (Thermo Fisher Scientific Inc., Massachusetts, USA) and 468 µl medium, and the blank 520 µl medium, were added carefully to three wells each. The well plate was then incubated for another 24 h ± 2 at 37 °C ± 1.

For the analysis, the specimens were carefully lifted from the cell layers and discarded before viewing them under the microscope. Then, 468 µl RPMI 1640 medium (Gibco by Thermo Fisher Scientific Inc., Massachusetts, USA) were added to each well, after turning off the lights, 52 µl PrestoBlue (PrestoBlue Cell Viability Reagent, Invitrogen by Thermo Fisher Scientific Inc., Massachusetts, USA) were added to each well. The well plate was wrapped in aluminum foil, to shield it from light, and then incubated for 2 hours at 37 °C ± 1. Then, from each well 100 µl were pipetted into three wells on a 96-well plate. Finally, the absorption was measured at 570 nm and 600 nm three times using the plate reader. The absorption values for each group were determined in the same way as described for the indirect cytotoxicity test.

All handling of medium, specimen, and cells was performed under a sterile bench (HERASAFE by Thermo Fisher Scientific Inc., Massachusetts, US).

### Statistical analyses

The sample size was determined based on similarly structured studies. The data were statistically analyzed using SPSS Statistics 25 (IBM). Each investigated property was used as the dependent variable in a one-way ANOVA. for multiple comparisons and significant differences between groups, a post hoc Tukey HSD test was conducted. The significance threshold was set at a p-value of less than 0.05.

## Results

### Physical characterization

**Fig. 3:**
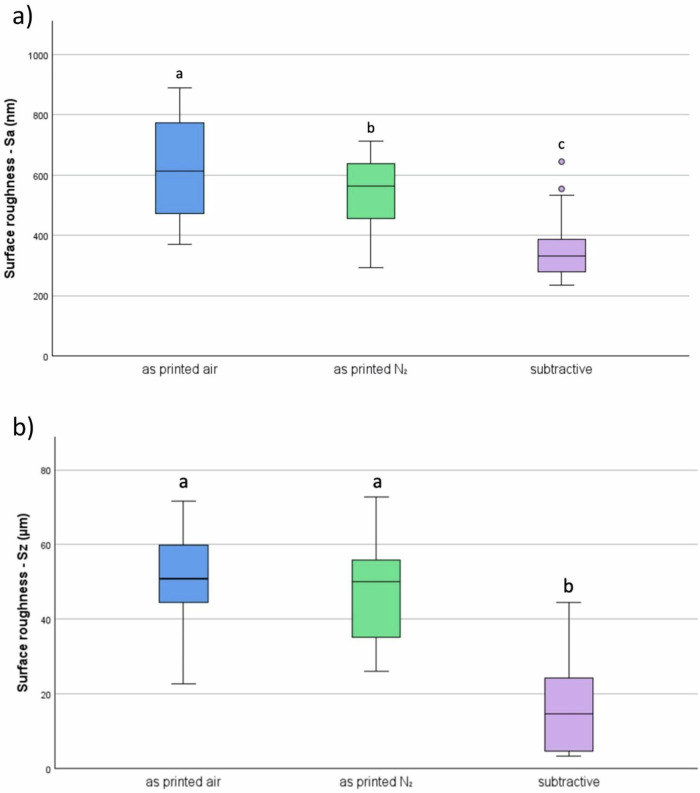
Surface roughness (**a**) Sa – arithmetic mean height and (**b**) Sz – maximum height of the different sample groups, expressed by the resulting surface roughness after the polishing protocol. Different lowercase letters indicate significant differences between groups *(p* < 0.05)

The results of polishability, flexural strength, and Vickers hardness are shown in Fig. 3a,b, as well as 4a and b. Statistically significant differences between the nitrogen and non-nitrogen groups were found only in Vickers hardness and polishability related to Sa, while flexural strength and polishability related to Sz did not differ significantly.

Regarding polishability (Fig. 3a, b), the subtractive CediTEC group demonstrates a statistically significant lower maximum height Sz after polishing compared to the opposing additive groups, which did not show significant differences among themselves. Meanwhile, the arithmetic mean of the heights of the selected area Sa revealed significantly different results across all groups.

Concerning flexural strength, the subtractive group also shows a lower MPa value than all four additively manufactured groups, which did not differ significantly from each other. All specimens of the additive groups broke in the process of the test, showing more brittle fracture, whereas the subtractively manufactured specimens did not break, but showed plastic deformation.

**Fig. 4:**
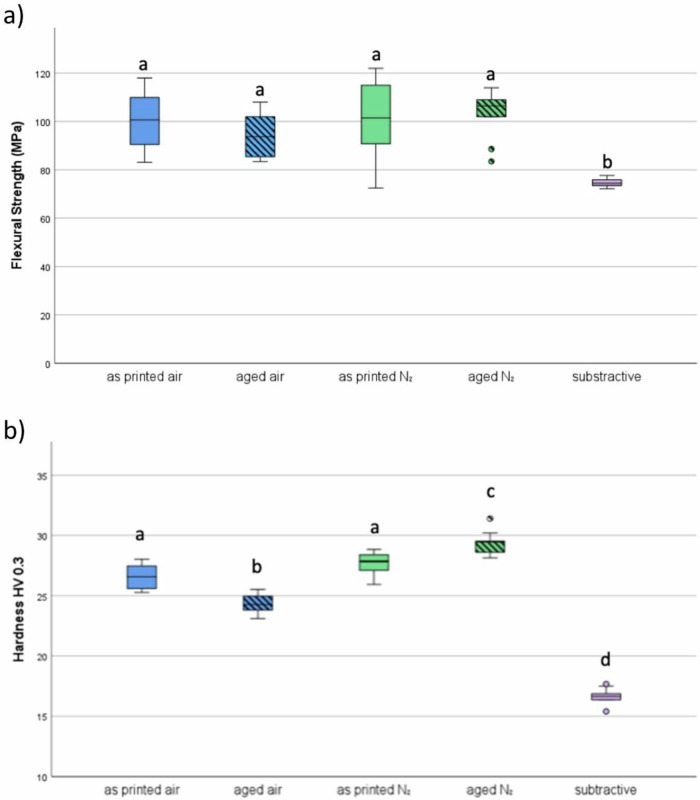
**a** flexural strength of the different groups **b** Vickers hardness. Different lowercase letters indicate significant differences between treatments (*p* < 0.05)

Concerning Vickers hardness, the subtractive group shows a significant difference in comparison to all additively manufactured groups, achieving a lower hardness. Within the additively manufactured groups, the following succession of hardness values from lowest to highest was measured: in air, aged < in air, as printed < in nitrogen, as printed < in nitrogen, aged. The differences are statistically significant between both age groups and all other additively manufactured groups. Additionally, the differences between the two as-printed groups are not significant when compared to each other, but they are significant when compared to all other groups Fig. 4.

### Degree of conversion

**Fig. 5:**
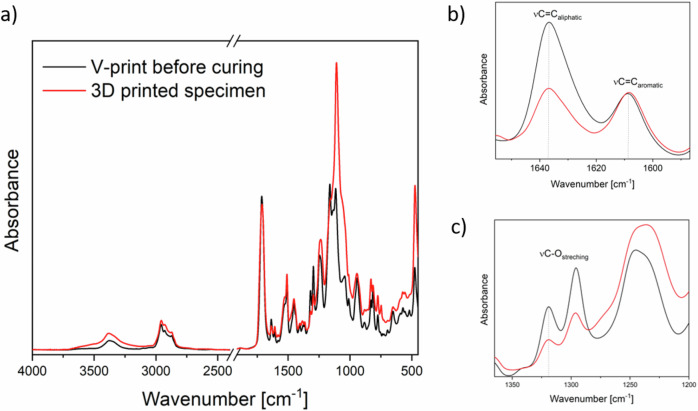
Normalized ATR spectra of the V-print dentbase before polymerization (black) and of the 3D printed specimen without post-processing step. **a** Overall spectrum, (**b**) 1600-1650 cm^−1^ and (**c**) ranges used for the DC calculation. Spectra were normalized by peak absorbance at 1637 cm^−1^

For calculating the degree of conversion FTIR spectra of the uncured resin and cured samples were measured and compared (Fig. 5) with two different techniques. We examined changes in the bands located at 1637 and 1320 cm^−1^, attributed to νC=C and νC-O of methacrylate, respectively, using the νC=C peak of the phenyl groups of BIS-EMA at 1608 cm^-1^ as a reference (Fig. 5b, c).

**Fig. 6:**
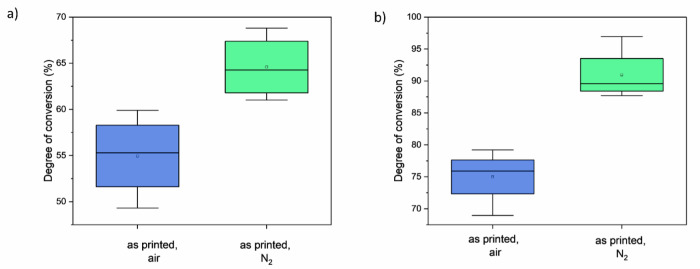
Degree of conversion of methacrylate groups of V-print dentbase in 3D printed specimens, with post-processing in air or in nitrogen atmosphere. **a** DC calculated from height of νC = C peak at 1637 cm^−1^, and **b** νC-O peak at 1320 cm^−1^

Figure 6 illustrates the DC obtained using both techniques. Although the absolute values differ depending on the chosen method, the trends are very similar for both DC calculation methods when comparing samples with different post-processing methods. There is no statistically significant difference in DC between non-post-cured and oxygen-cured samples; however, curing in a nitrogen atmosphere led to a significant increase in DC. Depending on the applied method, the DC is 10–16% higher for specimens post-cured in nitrogen.

### Biocompatibility testing

**Fig. 7:**
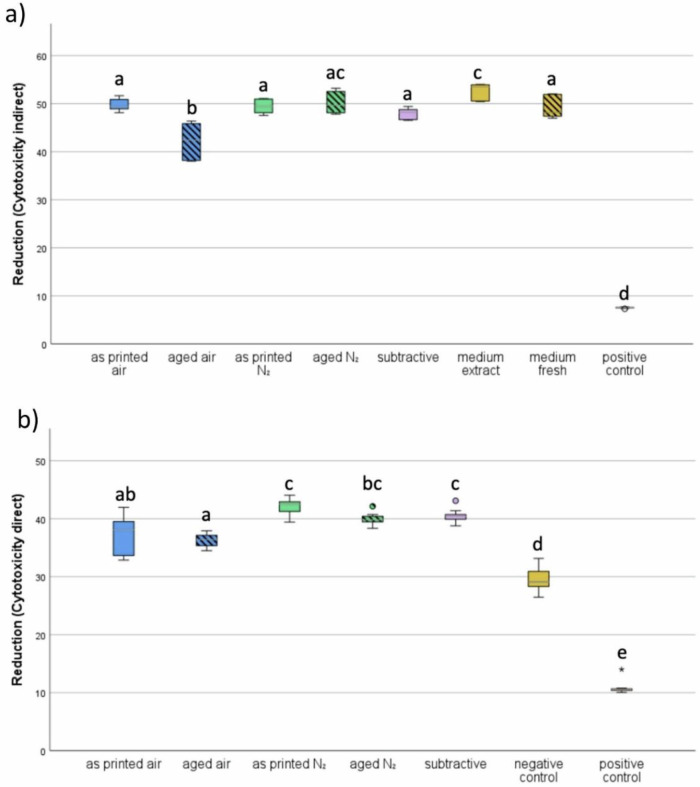
Results of cytotoxicity. **a** indirect test method (**b**) direct test method. Different lowercase letters indicate significant differences between treatments (*p* < 0.05)

Concerning cytotoxicity, the indirect test shows groups ‘aged air’ and ‘positive control’ have significant differences from all other groups, as represented in Fig. 7a. The group ‘medium extract’ shows significant differences from all groups except for ‘aged N₂’. Within all groups, the following succession of reduction levels was measured from highest to lowest: medium extract > aged N₂ > as printed air > as printed N₂ > medium fresh > subtractive > aged air > positive control.

**Fig. 8:**
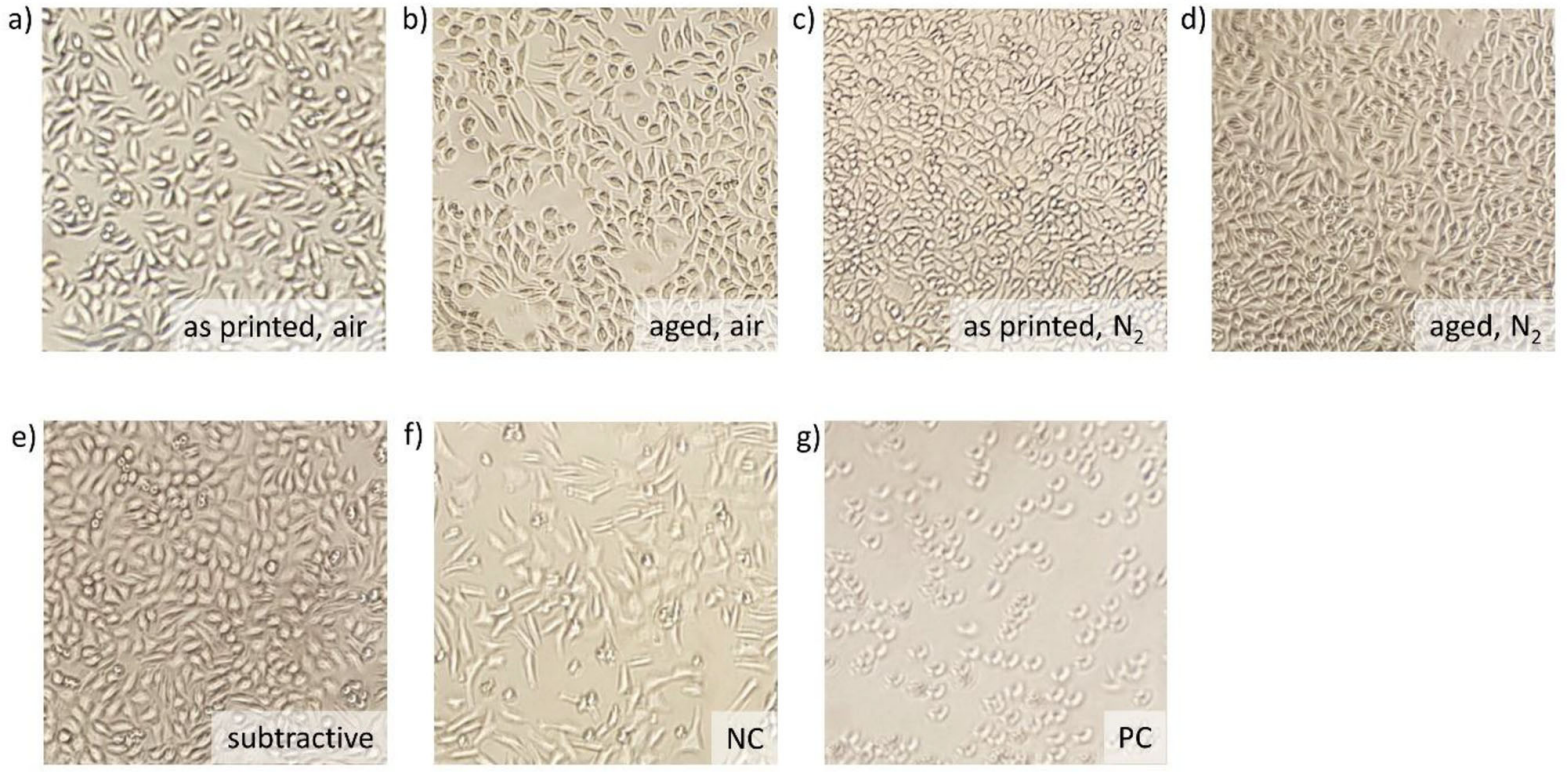
Cells after the direct cytotoxicity test

The direct test (Fig. 7b) shows the same result for the positive control group but also indicates that the group with nitrogen post-processing and no aging has the least cytotoxicity, followed by the subtractively manufactured group and the group using nitrogen post-processing and aging. The third least cytotoxic groups are the two groups that were post-processed without nitrogen. The lowest scores, except for the positive control group, were measured in the titanium group, which also shows significant differences from all other groups. Except for control groups, the group printed air shows significantly different values from groups ‘as printed N₂’ and ‘subtractive’; ‘aged air’ shows significantly different results from all groups except ‘as printed air’; ‘printed N₂’ shows significant differences from ‘as printed air’ and ‘aged air’; ‘aged N₂’ only shows significant differences from ‘aged air’. As shown in Fig. 8 below, the cells treated with DMSO are round and not stuck to the bottom of the cell culture flask, like the cells of all other groups.

## Discussion

### Findings of experiments

Statistically significant differences were observed in biocompatibility and surface hardness when comparing post-curing in air versus nitrogen atmospheres. Nitrogen post-curing led to improved surface properties and higher degree of conversion (DC), as confirmed by ATR-FTIR analysis. This supports the hypothesis that nitrogen atmosphere during post-processing reduces cytotoxicity without compromising mechanical integrity. Increased DC leads to fewer residual monomers on unpolished surfaces, improving biocompatibility [13, 14].

The process of photopolymerization in methacrylates involves the opening of a carbon-carbon double bond, which can be observed in the infrared spectrum as a reduction in the peaks at 1630-1640 cm^−1^ (νC=C) and 1295-1320 cm^-1^ (νC-O) (Fig. 5b, c) [19]. While the 1635 cm^−1^ band is commonly used to quantify DC, this region can be affected by O-H bending from water and shifts in carbonyl peaks. Recent studies suggest that bands around 1295–1320 cm^−1^ provide more consistent results [19–22]. Regardless of the spectral region used, our data showed that post-curing in nitrogen atmosphere significantly increased DC, although no surface reached 100% conversion due to limited radical mobility beyond the gel point [23]. This observation is consistent with oxygen’s known role as a radical scavenger [24].

Improved surface hardness after nitrogen post-curing likely results from a denser polymer network and reduced oxygen inhibition [15, 16, 22]. However, since this effect is limited to the outermost ~20 µm of the material [16], polishability is not negatively impacted. Flexural strength was not significantly affected, likely due to the minimal volume fraction influenced by surface curing.

Among tested materials, the subtractive material CediTEC showed statistically significantly lower mechanical performance, and less brittleness, demonstrated by plastic deformation rather than fracture. Plastic deformability is advantageous in dental prosthetic materials because it reduces the risk of sudden material failure. Unlike brittle materials, which tend to fracture abruptly under stress, ductile materials can absorb and redistribute mechanical loads by undergoing slight deformation. This behavior is especially beneficial under masticatory forces, where localized stress peaks may otherwise lead to cracks or fractures. Additionally, plastic deformability allows for better stress accommodation in thin-walled or complex geometries and provides a level of tolerance during clinical adjustment and fitting.

The results from both indirect (Fig. 7a) and direct (Fig. 7b) cytotoxicity tests indicate that the post-curing atmosphere significantly influences the biocompatibility of 3D-printed resin materials. Specimens post-cured in a nitrogen atmosphere (“as printed N₂“ and “aged N₂“) consistently showed higher reduction of resazurin—reflecting higher cell viability—compared to those post-cured in air.

In the indirect test (Fig. 7a), the “aged air” group showed significantly lower biocompatibility than all other test groups, including both nitrogen-cured and subtractive controls. The “aged N₂” group showed comparable performance to “as printed N₂” and “subtractive,” suggesting that nitrogen post-curing not only improves biocompatibility but also maintains it after artificial aging.

In the direct test (Fig. 7b), similar trends were observed. While the difference between groups was somewhat less pronounced, nitrogen-cured groups still showed higher biocompatibility compared to “aged air” and clearly outperformed the negative and positive controls. The “as printed air” group also performed better than “aged air,” indicating that air-curing may accelerate the release or persistence of cytotoxic residual monomers during aging. The negative control group values weren’t exactly zero because the glucose in the medium also results in reduction of Resazurin [20].

Overall, the data support the hypothesis that nitrogen post-curing enhances the degree of conversion, thereby reducing the concentration of unreacted, cytotoxic monomers on the material surface. This effect is critical, particularly for intraoral applications where unpolished surfaces remain in prolonged contact with soft tissues. The comparable performance of subtractive specimens and nitrogen-cured printed materials further underscores the potential of optimized additive manufacturing workflows to match or even exceed the biocompatibility of traditional materials.

### Clinical interpretation

A study by Jin et al. [14] showed that reducing residual monomers by an enhanced washing process decreased the cytotoxicity of 3D-printed resin materials, benefiting their biocompatibility. The free radicals, as they are found in the free monomers, are cytotoxic [25], which indicates that a reduction, as can be achieved by omitting the oxygen inhibition layer [16, 26, 27] by light curing under a nitrogen atmosphere, benefits the biocompatibility of 3D-printed resins.

Since all dentures include a surface that is not polished or whose top layers are otherwise ablated, it is crucial to reduce the cytotoxicity as much as possible, for instance, via post-processing. This is especially important since this surface is in direct contact with the oral mucosa for a long period. Regarding this study, it could be shown that light-curing 3D-printed resin for denture bases in a nitrogen-flooded chamber benefits the materials’ biocompatibility without compromising their mechanical properties.

Although it is helpful to follow manufacturers’ instructions, such as which solvent solutions and post-curing times should be used, since these affect the mechanical properties [8], some alterations of these steps might result in enhanced qualities, such as higher biocompatibility and surface hardness, as shown by this study.

As the mentioned study by Oh et al. [10] showed, higher washing solution temperatures and washing times can increase the biocompatibility of 3D-printed resins. But while in the said study the mechanical properties were impaired by those alterations of the post-processing, our study facilitates higher biocompatibility with even higher surface hardness and unaffected flexural strength.

Increasing biocompatibility without deteriorating the surface hardness, flexural strength, and polishability is of very high interest when it comes to medical products such as dentures. As the digital manufacturing process becomes more and more common in the dental field, determining the optimal procedure is not only desirable but vital to ensure the best possible patient care.

Therefore, when printing materials that will be in contact with gingival tissue, it is advisable to light-cure the objects in a nitrogen-flooded chamber to enhance biocompatibility.

### Study design

Although many studies [28–30] have employed the same method for artificial aging, the process is simplified and shortened compared to the natural aging process. Therefore, results may vary from actual outcomes.

This study only evaluated the biocompatibility of specimens in an in vitro environment, leading to limitations regarding all environmental influences and stresses affecting the oral cavity. Additionally, the cells used are not human mucosa cells, which may have yielded different results.

Concerning the polishability, a potential source of error is the polishing being performed by a human, as it cannot be guaranteed that each specimen is polished with the exact same pressure and motion. Human error limits the possible accuracy of this portion of the study.

To gain further scientific insights, these results should not only be compared with those regarding additional materials, but also, more mechanical properties should be investigated. The unexplored areas and the growing demand for better digital workflows in the production of dental appliances highlight the need for further research in this domain. Only by conducting such research can we achieve better, quicker, and more environmentally friendly medical care.

## Conclusion

- Degree of conversion, Cytotoxicity, and Vickers hardness are positively impacted by the use of nitrogen during the post-processing of 3D-printed resin.
- The flexural strength and polishability are not affected by the use of nitrogen as opposed to air during the post-processing of 3D-printed resin.
- If higher biocompatibility and surface hardness are desired, dentures, which are manufactured using 3D-printed resins, such as V-Print dentbase by Voco GmbH, should be post-processed using nitrogen-flooded light curing chambers.

## References

[1] Anderson J, Wealleans J, Ray J. Endodontic applications of 3D printing. Int Endod J. 2018;51:1005–18. 29486052 10.1111/iej.12917

[2] Dawood A, Marti Marti B, Sauret-Jackson V, Darwood A. 3D printing in dentistry. Br Dent J. 2015;219:521–9. 26657435 10.1038/sj.bdj.2015.914

[3] Daher R, Ardu S, di Bella E, Krejci I, Duc O. Efficiency of 3D printed composite resin restorations compared with subtractive materials: Evaluation of fatigue behavior, cost, and time of production. J Prosthet Dent. 2024;131:943–50. 36333176 10.1016/j.prosdent.2022.08.001

[4] Kurenov SN, Ionita C, Sammons D, Demmy TL. Three-dimensional printing to facilitate anatomic study, device development, simulation, and planning in thoracic surgery. J Thorac Cardiov Sur. 2015;149:973–9. 10.1016/j.jtcvs.2014.12.059 25659851

[5] Berglund G, Wisniowiecki A, Gawedzinski J, Applegate B, Tkaczyk TS. Additive manufacturing for the development of optical/photonic systems and components. Optica. 2022;9:623–38.

[6] Ergül T, Güleç A, Göymen M. The use of 3D printers in orthodontics-a narrative review. Turk J Orthod. 2023;36:134–43. 37346463 10.4274/TurkJOrthod.2022.2021.0074 PMC10318848

[7] Revilla-León M, Özcan M. Additive manufacturing technologies used for processing polymers: current status and potential application in prosthetic dentistry. J Prosthodont. 2019;28:146–58. 29682823 10.1111/jopr.12801

[8] Finck NS, Fraga MAA, Correr AB, Dalmaschio CJ, Rodrigues CS, Moraes RR. Effects of solvent type and UV post-cure time on 3D-printed restorative polymers. Dent Mater. 2024;40:451–7. 38129193 10.1016/j.dental.2023.12.005

[9] Jersovaite J, Sarachovaite U, Matulaitiene I, Niaura G, Baltriukiene D, Malinauskas M. Biocompatibility enhancement via post-processing of microporous scaffolds made by optical 3D printer. Front Bioeng Biotech. 2023;11:1167753. 10.3389/fbioe.2023.1167753 PMC10130666 37122855

[10] Oh R, Lim JH, Lee CG, Lee KW, Kim SY, Kim JE. Effects of washing solution temperature on the biocompatibility and mechanical properties of 3D-Printed dental resin material. J Mech Behav Biomed. 2023;143:105906. 10.1016/j.jmbbm.2023.105906 37178635

[11] Alshamrani AA, Raju R, Ellakwa A. Effect of printing layer thickness and postprinting conditions on the flexural strength and hardness of a 3d-printed resin. Biomed Res Int. 2022;2022:8353137. 35237691 10.1155/2022/8353137 PMC8885203

[12] Tangpothitham S, Pongprueksa P, Inokoshi M, Mitrirattanakul S. Effect of post-polymerization with autoclaving treatment on monomer elution and mechanical properties of 3D-printing acrylic resin for splint fabrication. J Mech Behav Biomed. 2022;126:105015. 10.1016/j.jmbbm.2021.105015 34896766

[13] Raszewski Z, Nowakowska-Toporowska A, Nowakowska D, Wieckiewicz W. Update on acrylic resins used in dentistry. Mini-Rev Med Chem. 2021;21:2130–7. 33634758 10.2174/1389557521666210226151214

[14] Jin G, Gu HN, Jang M, Bayarsaikhan E, Lim JH, Shim JS, Lee KW, Kim JE. Influence of postwashing process on the elution of residual monomers, degree of conversion, and mechanical properties of a 3D printed crown and bridge materials. Dent Mater. 2022;38:1812–25. 36192277 10.1016/j.dental.2022.09.017

[15] Suh BI. Oxygen-inhibited layer in adhesion dentistry. J Esthet Restor Dent. 2004;16:316–23. 15726800 10.1111/j.1708-8240.2004.tb00060.x

[16] Panchal AC, Asthana G. Oxygen inhibition layer: a dilemma to be solved. J Conserv Dent. 2020;23:254–8. 33551595 10.4103/JCD.JCD_325_19 PMC7861070

[17] Zinelis S, Panayi N, Polychronis G, Dionysopoulos D, Papageorgiou SN, Eliades T. Effect of nitrogen atmosphere during 3D printing on mechanical properties of orthodontic aligners. Eur J Oral Sci. 2025;133:e70008. 39984176 10.1111/eos.70008

[18] Chen L, Li D, Zhou J, Lin WS, Tan J. Duplicating complete dentures with conventional and digital methods: comparisons of trueness and efficiency. Dent J (Basel). 2022;10:35. 35323238 10.3390/dj10030035 PMC8947193

[19] Delgado AHS, Young AM. Methacrylate peak determination and selection recommendations using ATR-FTIR to investigate polymerisation of dental methacrylate mixtures. PLoS One. 2021;16:e0252999. 34106972 10.1371/journal.pone.0252999 PMC8189511

[20] Walters NJ, Xia WD, Salih V, Ashley PF, Young AM. Poly(propylene glycol) and urethane dimethacrylates improve conversion of dental composites and reveal complexity of cytocompatibility testing. Dent Mater. 2016;32:264–77. 26764174 10.1016/j.dental.2015.11.017

[21] Sullivan B, Kalliecharan D, Kostylev I, Earle G, Stansbury JW, Price RB, Labrie D. Photo-polymerization kinetics of a dental resin at a high temporal resolution. J Mech Behav Biomed Mater. 2021;124:104884. 34638087 10.1016/j.jmbbm.2021.104884

[22] Rajan G, Raju R, Jinachandran S, Farrar P, Xi JT, Prusty BG. Polymerisation shrinkage profiling of dental composites using optical fibre sensing and their correlation with degree of conversion and curing rate. Sci Rep-Uk. 2019;9:3162. 10.1038/s41598-019-40162-z PMC6395615 30816275

[23] Pianelli C, Devaux J, Bebelman S, Leloup G. The micro-Raman spectroscopy, a useful tool to determine the degree of conversion of light-activated composite resins. J Biomed Mater Res. 1999;48:675–81. 10490681 10.1002/(sici)1097-4636(1999)48:5<675::aid-jbm11>3.0.co;2-p

[24] Decker C, Jenkins AD. Kinetic approach of oxygen inhibition in ultraviolet- and laser-induced polymerizations. Macromolecules. 1985;18:1241–4.

[25] Juranova J. Illuminating the cellular and molecular mechanism of the potential toxicity of methacrylate monomers used in biomaterials. Drug Chem Toxicol. 2020;43:266–78. 30607995 10.1080/01480545.2018.1488860

[26] Shawkat ES, Shortall AC, Addison O, Palin WM. Oxygen inhibition and incremental layer bond strengths of resin composites. Dent Mater. 2009;25:1338–46. 19595445 10.1016/j.dental.2009.06.003

[27] Finger WJ, Lee KS, Podszun W. Monomers with low oxygen inhibition as enamel/dentin adhesives. Dent Mater. 1996;12:256–61. 9002844 10.1016/s0109-5641(96)80032-7

[28] Deng D, Yang H, Guo J, Chen X, Zhang W, Huang C. Effects of different artificial ageing methods on the degradation of adhesive-dentine interfaces. J Dent. 2014;42:1577–85. 25257825 10.1016/j.jdent.2014.09.010

[29] Yun XF, Li W, Ling C, Fok A. Effect of artificial aging on the bond durability of fissure sealants. J Adhes Dent. 2013;15:251–8. 23534030 10.3290/j.jad.a29014

[30] Heintze SD, Ilie N, Hickel R, Reis A, Loguercio A, Rousson V. Laboratory mechanical parameters of composite resins and their relation to fractures and wear in clinical trials-A systematic review. Siegward Dent Mater. 2017;33:E101–14. 27993372 10.1016/j.dental.2016.11.013

